# Differential selective pressure alters rate of drug resistance acquisition in heterogeneous tumor populations

**DOI:** 10.1038/srep36198

**Published:** 2016-11-07

**Authors:** Daphne Sun, Simona Dalin, Michael T. Hemann, Douglas A. Lauffenburger, Boyang Zhao

**Affiliations:** 1 Department of Biological Engineering, Massachusetts Institute of Technology, Cambridge, MA 02139, USA; 2 Department of Biology, Massachusetts Institute of Technology, Cambridge, MA 02139, USA; 3 The David H. Koch Institute for Integrative Cancer Research, Cambridge, MA 02139, USA; 4 Computational and Systems Biology Program, Massachusetts Institute of Technology, Cambridge, MA 02139, USA.

## Abstract

Recent drug discovery and development efforts have created a large arsenal of targeted and chemotherapeutic drugs for precision medicine. However, drug resistance remains a major challenge as minor pre-existing resistant subpopulations are often found to be enriched at relapse. Current drug design has been heavily focused on initial efficacy, and we do not fully understand the effects of drug selective pressure on long-term drug resistance potential. Using a minimal two-population model, taking into account subpopulation proportions and growth/kill rates, we modeled long-term drug treatment and performed parameter sweeps to analyze the effects of each parameter on therapeutic efficacy. We found that drugs with the same overall initial kill may exert differential selective pressures, affecting long-term therapeutic outcome. We validated our conclusions experimentally using a preclinical model of Burkitt’s lymphoma. Furthermore, we highlighted an intrinsic tradeoff between drug-imposed overall selective pressure and rate of adaptation. A principled approach in understanding the effects of distinct drug selective pressures on short-term and long-term tumor response enables better design of therapeutics that ultimately minimize relapse.

Recent high-throughput sequencing studies revealed extensive intratumoral heterogeneity in patients^1^,^2^. Sequencing of clinical data and quantitative modeling approaches have shown tumor clonal evolution to be highly dynamic, with minor subclones oftentimes selected for during drug treatment^3^,^4^,^5^,^6^,^7^,^8^. Such drug-imposed selective pressures can affect the trajectories of evolution, and further, can be in competition with various other processes, such as background mutation rates, fitness of the resulting mutations, and clonal cooperativity/interference^9^,^10^,^11^,^12^.

Given this evidence, it follows that tumor heterogeneity and dynamics need to be considered during the diagnosis and treatment of cancer. Both stochastic^13^,^14^,^15^,^16^,^17^,^18^,^19^,^20^,^21^,^22^,^23^,^24^,^25^,^26^ and deterministic^27^,^28^ approaches have been used to model the clonal dynamics of the initiation, progression, and development of heterogeneity in cancers. Other studies have used similar mathematical/computational approaches to model tumor clonal dynamics under drug treatment^5^,^6^,^7^,^8^,^29^,^30^ and design optimal drug combinations and/or dosing schedules in the regime of these complex dynamics^31^,^32^. We have previously used a computational approach to rationally design drug combinations in the context of tumor heterogeneity^33^. The objective in the optimization model was to find drug combinations that can best maximize the initial killing given a particular heterogeneous tumor composition. While maximizing initial tumor killing constitutes one goal in clinical therapeutic design, ultimately delaying the onset of drug resistance is critical for prolonged survival. Several studies have examined unconventional regimens that may influence overall survival^34^,^35^. However, the question remains as to how exactly overall drug-imposed selective pressure affects tumor dynamics and long-term drug resistance potential.

Here, we perform a systematic computational analysis to interrogate the effects of different drug-imposed selective pressures on long-term therapeutic outcome. We are especially interested in the case where two different regimens can have the same initial efficacy on the overall tumor, but differential drug-imposed selective pressures on the individual subpopulations may lead to significantly different drug sensitivities in the long-term. Systematic sampling of the parameter space brought additional insight into the dependencies of growth rates, kill rates, and subpopulation proportions on potential predictability of therapeutic outcome, and intrinsic tradeoffs in therapeutic design.

## Results

We focused our analysis in the regime of large population size (e.g. at times of diagnosis), and assumed that the population is well-mixed and contains pre-existing resistant subpopulations. While stochastic drift and background mutation rates are also important driving forces in clonal evolution and resistance^12^, we are interested here in regimes where there already exists resistant subpopulation(s) prior to drug treatment. This has been clinically observed in a number of studies^4^,^6^,^7^,^8^, where, while the pre-existing resistant cells are in the minority, the subpopulation size is still substantial enough that selection is the dominating driving process in resistance^36^. Thus, we can assume a sufficient initial subpopulation size such that tumor dynamics can be modeled as deterministic processes, using ordinary differential equations (ODEs). Several parameters are of relevance. These include growth rates and drug killing rates of each subpopulation, overall growth and kill rates of the tumor, and the relative size of each subpopulation in the initial tumor (see Methods for details of the mathematical model).To effectively make comparisons across different drug regimens under the influence of these parameters, we required a common reference condition. Therefore, we ensured the same initial overall tumor size at the time of treatment and initial tumor reduction following the first treatment cycle between drug regimens. As such, this enabled us to compare various regimens, such as cases where two regimens result in the same overall initial tumor reduction, but cause vastly different resistance potentials due to differential killing of the subpopulations. We will refer to a treatment with differential killing of subpopulations as ‘asymmetric treatment’, and equal killing as ‘symmetric treatment’, for the remainder of this paper.

### Differential Selective Pressure Affects Therapeutic Outcome

**Figure 1:**
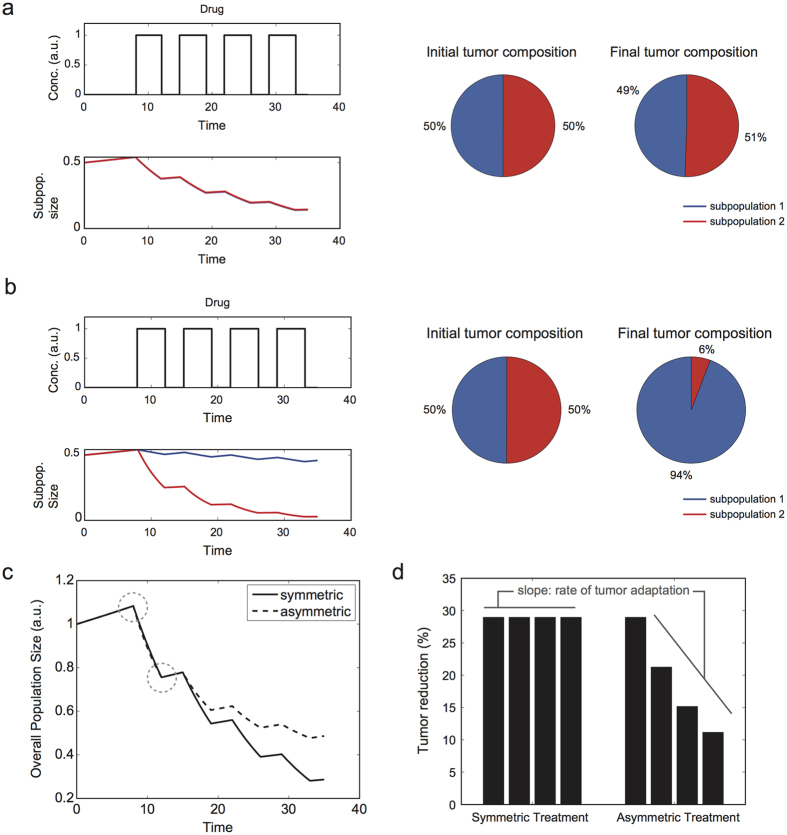
Representative tumor kinetics under regimens with distinct selective pressures on subpopulations. (**a**) Tumor kinetics of a minimal two-subpopulation tumor under a ‘symmetric’ regimen. Here, the drug has approximately equal selective pressure (i.e. killing) on the two subpopulations. Multiple rounds of drug treatment results in nearly the same tumor composition (with respect to the ratio of the two subpopulations). (**b**) Kinetics under an ‘asymmetric’ regimen with differential killing of the subpopulations, resulting in changes to the final tumor composition over multiple rounds of drug treatment. (**c,d**) Comparison of the overall tumor kinetics under symmetric and asymmetric treatment, illustrating that although the overall killing is the same after the first initial dose, asymmetric treatment results in higher tumor burden in the long-term (**c**) and reduced drug sensitivity (**d**).

We first examined a single scenario contrasting a symmetric treatment, with equal subpopulation kill, and an asymmetric treatment, with unequal subpopulation kill. Upon successive symmetric treatments, we expectedly observed comparable tumor compositions at the initial and final time points (Fig. 1a, as a representative example of approximately symmetric killing). In contrast, an asymmetric treatment led to the outgrowth of one subpopulation upon multiple rounds of treatment (Fig. 1b). These results are intuitive. However, the important metrics of relevance for our later analyses are the following. The first is with respect to overall tumor size: in accordance with our constraints, the two regimens (i.e. symmetric vs asymmetric) had the same initial tumor burden and tumor reduction following a single round of treatment. As such, the initial efficacy of both regimens are comparable. Nevertheless, repetitive treatments led to a divergence in efficacy, with symmetric treatment having a smaller final tumor burden (Fig. 1c). This divergence in efficacy is also apparent when we examine other metrics, such as the percent tumor reduction after each round of treatment (Fig. 1d). In addition, by taking the slope of the percent tumor reduction values for successive doses, we can determine the rate of change in tumor sensitivity. In other words, the greater the decrease in tumor reduction after each round of treatment, the more negative the rate of change in tumor sensitivity. Taking the absolute value of this metric can be thought of as the rate of adaptation, which refers to how quickly the population composition changes (and thus responds to therapy) upon successive rounds of drug treatment. Because the tumor either retains the same or diminishing drug sensitivity in our analyses, we use the term ‘rate of adaptation’ for the rest of this paper to refer to the rate of acquisition of resistance.

### Computational Sampling to Identify Mapping of Distinct Parameter Regions to Therapeutic Outcome

Using these aforementioned metrics (i.e. overall tumor size and rate of adaptation), we next studied how varying parameters and particular parameter regions affect therapeutic outcomes. Our ODE system of equations is fully defined by the following set of parameters: overall growth rate (*k*_*s*_), overall kill rate (*α*_*s*_), growth rate (*k*_1_) and kill rate (*α*_1_) of subpopulation 1, and the initial fraction of each subpopulation (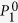 and 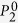). Given a defined set of values for these parameters, we can correspondingly determine growth rate (*k*_2_) and kill rate (*α*_2_) of the second subpopulation (see Methods for details on mathematical derivations, proofs, and constraints analyses).

**Figure 2:**
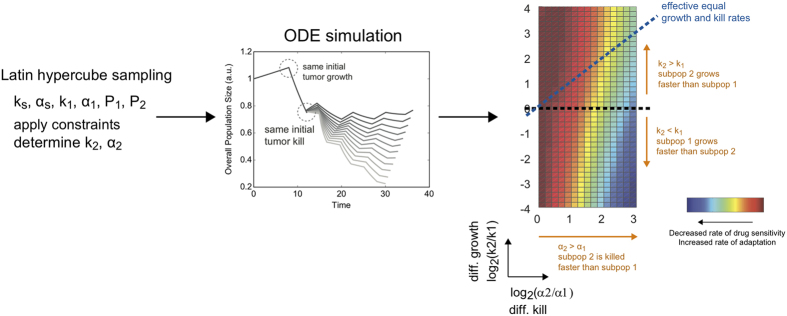
Analysis procedure to identify parameter regimes with high/low therapeutic efficacy. Latin hypercube sampling was performed on the following parameters: overall growth rate (*k*_*s*_), overall kill rate (*α*_*s*_), growth rate (*k*_1_) and kill rate (*α*_1_) of subpopulation 1, and the initial fraction of each subpopulation (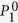 and 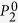). See Supplementary Table S1 for the parameter range used. Provided with the constraint of equal tumor burden and tumor reduction following first round of treatment, we derived the corresponding growth rate (*k*_2_) and kill rate (*α*_2_) of subpopulation 2. For each given parameter set, we ran the ODE simulations and derived therapeutic metrics (tumor size, percent tumor reduction, and rate of change in tumor sensitivity). This was visualized on a two-dimensional heatmap of differential growth (*k*_2_/*k*_1_) versus differential killing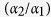.

We performed Latin hypercube sampling of the parameter sets (Fig. 2). We focused our analysis mainly on the effects of differential growth rate and kill rate on our therapeutic metrics, as influenced by different overall growth rates, kill rates, and subpopulation proportions. This analysis can be best visualized on a two-dimensional plot of differential kill rate (*α*_2_/*α*_1_) versus differential growth rate (*k*_2_/*k*_1_). Traversing along the abscissa toward the right, we increase the differential killing of the tumor subpopulations (i.e. differential selective pressure). On the other hand, values deviating up or down from the center of the ordinate axis correspond to differential growth rates of the subpopulations. This visualization also suggests the existence of a diagonal line corresponding to an ‘effective’ equal growth and kill rate as we transverse in this parameter space (dotted diagonal line in Fig. 2). Here, the differential growth rate is balanced by the differential killing rate such that the net effect is symmetric growth and killing.

#### Increase in overall selective pressure broadens parameter range of adaptation rate

**Figure 3:**
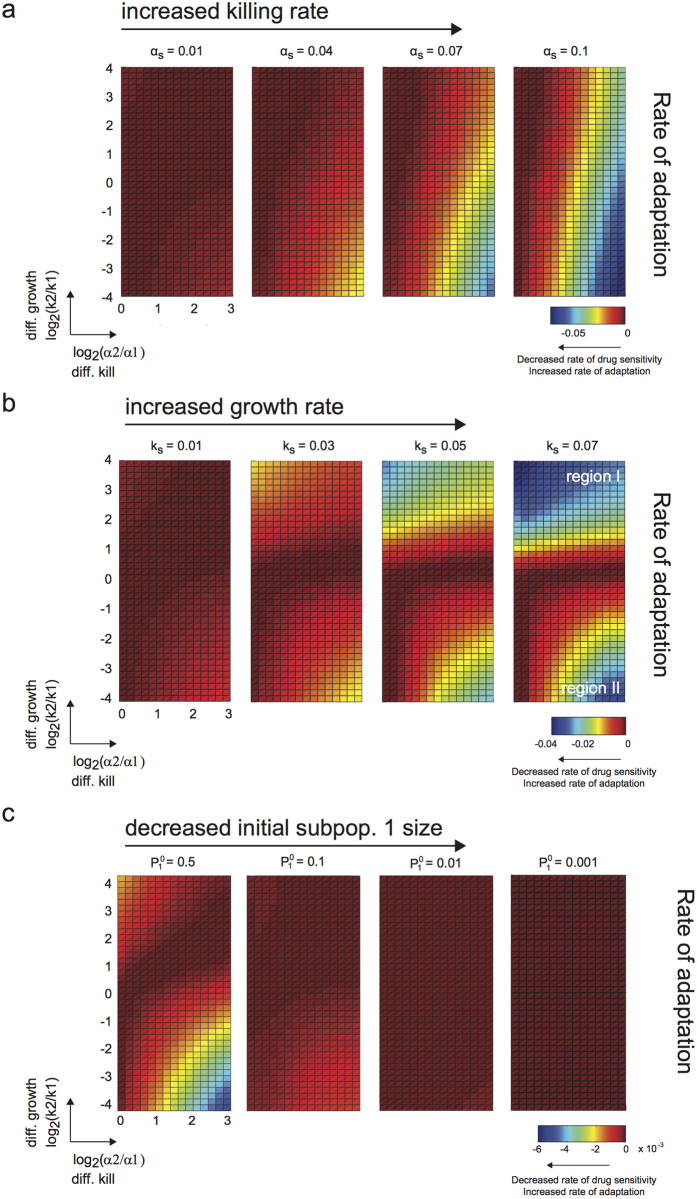
Distinct parameter regimes influence rate of adaptation and acquisition of resistance. (**a–c**) Heatmaps showing rate of adaptation of tumor population to drug upon multiple rounds of drug treatment, given the specified model parameters (killing rates, growth rates, and initial subpopulation proportions). More accurately, the rate of change in drug sensitivity is shown, but this is directly related to the rate of adaptation. The abscissa axis is the ratio of the kill rates on the two subpopulations, and represents the extent of differential killing, or in other words the bias in killing of one subpopulation over the other. The ordinate axis is the ratio of growth rates, and represents the extent of differential growth between the two subpopulations. (**a**) Each subpanel shows the rate of adaptation corresponding to a given overall killing rate, and is ordered from left to right in increasing overall kill. Increased overall kill results in a more pronounced region of high rate of adaptation. Growth rate (*k*_*s*_) was fixed at 0.01 hr^−1^. (**b**) Similar layout as shown in (**a**), but with increasing overall growth rates. Increased overall growth rate results in two distinct pronounced regions of increased rate of adaptation. Killing rate (*α*_*s*_) was fixed at 0.01 hr^−1^. (**c**) Similar layout as shown in (**a**), but with decreasing initial subpopulation proportion.

Using this analysis procedure and visualization, we first examined the effects of varying overall killing rate (*α*_*s*_) on long-term therapeutic efficacy. We observed that the region of high rate of adaptation (i.e. more negative rate of change in tumor sensitivity, colored blue in the heatmap in Fig. 3a) broadens with increased overall kill, particularly along the differential growth rate axis. This suggests that when we increase the overall selective pressure on the tumor population, the rate of adaptation becomes less dependent on the differences in growth rates between subpopulations. In addition, increased differential killing of the subpopulations led to increased rate of adaptation (Fig. 3a and Supplementary Fig. S1a). Thus, differential drug-induced selective pressure plays a prominent role in predicting rate of adaptation (and thus development of resistance), and dominates over a broader range of differential growth rates of subpopulations as we increase the overall selective pressure on the tumor population.

Moreover, when we examined the corresponding final tumor size (Supplementary Fig. S2a), we observed that increasing the overall kill rate always leads to an overall decreased final tumor size. However, this comes with a cost of increasing the rate of adaptation in regions where there is significant difference in growth rates and kill rates (Supplementary Fig. S4). As such, there is a trade-off at higher overall selective pressures between overall tumor reduction and the rate of adaptation (and resistance acquisition).

#### Increase in overall growth rate affects rate of adaptation, contingent on directionality of relative fitness

Next, we explored how overall growth rate of a tumor population affects therapeutic efficacy. While overall growth rate is not a parameter we can modulate as easily in an experimental/clinical setting (as this can be an intrinsic proliferation rate of the tumor population), examination of this parameter gives us insight into the consequence of proliferation, as associated with a particular tumor type, on its resistance potential in light of the other parameters. Interestingly, we observed that the region of independence in the rate of adaptation no longer stretches along the ordinate axis, but rather broadens along the differential kill axis (region I in Fig. 3b and Supplementary Fig. S1b). This occurs only in the upper quadrants because of the direct competition between two parameters: the growth rate of subpopulation 2 (relative to subpopulation 1) and the killing rate of subpopulation 2 (relative to subpopulation 1). Increasing the overall growth rate of the tumor population amplifies the dominance in growth rate of subpopulation 2 over subpopulation 1, and thus minimizes the effect of differential selective pressure on the rate of adaptation. On the other hand, when the relative growth rate is in the opposite direction (i.e. subpopulation 1 has a higher fitness advantage over subpopulation 2), we expectably again observe the importance of differential drug selection on the rate of adaptation (region II in Fig. 3b and Supplementary Fig. S1b). Hence, when the proliferation of the tumor is high, and with preferential fitness advantage on the same subpopulation as preferentially targeted by the drug, we have the least ability to minimize the rate of adaptation and resistance through choosing drugs with desirable differential killing characteristics.

#### Initial subpopulation fractions affect magnitude in rate of adaptation across parameter regimes

Initial tumor composition can vary widely prior to treatment. We examined the effects of varying initial fractions of subpopulation 1 on therapeutic outcome. Here, we observed that decreasing the initial proportion of subpopulation 1 decreases the magnitude of rate of adaptation (Fig. 3c) and overall tumor size (Supplementary Fig. S2c) across the entire parameter space, but does not change the general topology of the distinct regions (Supplementary Figs S1c and S3c). Thus, differential selective pressure still plays a critical role in the rate of adaptation, but the timescale over which a resistant subpopulation arises and becomes apparent is affected by initial subpopulation sizes.

### Combinatorial effects of overall growth/kill rates constrain parameter regimes with high rate of adaptation

**Figure 4:**
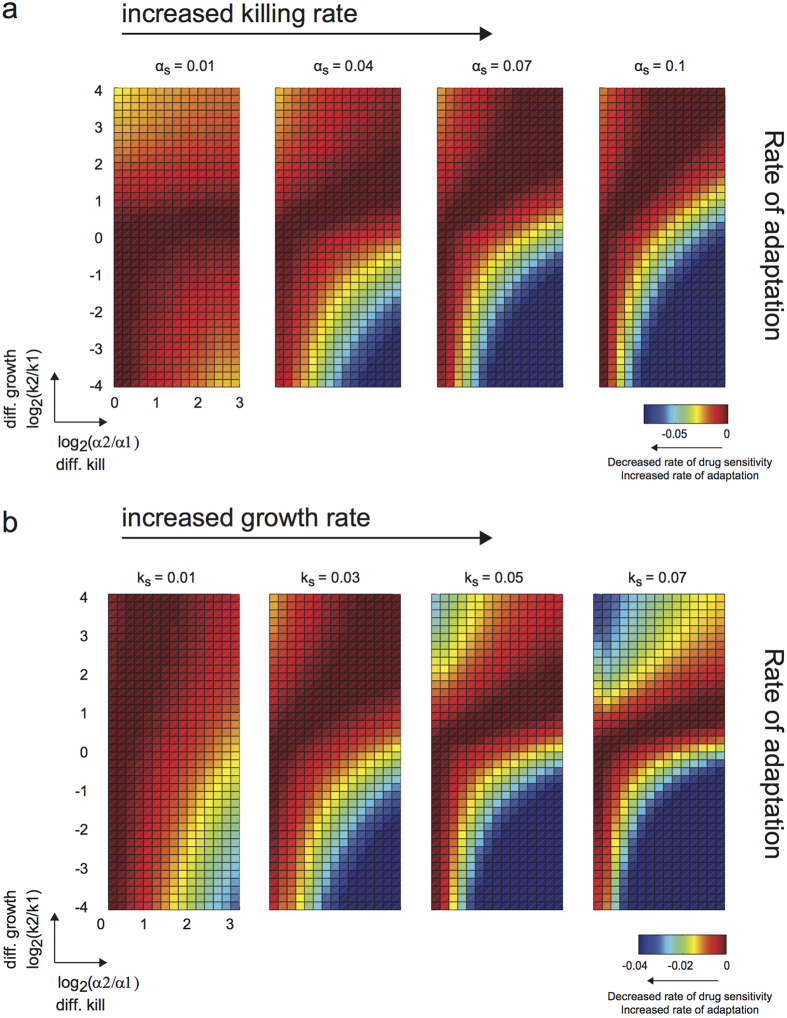
Combinatorial effects of overall growth and kill rates on parameter regimes with high/low rate of adaptation. (**a**) In contrast to Fig. 3a, the simulations here were performed at a higher basal overall growth rate (*k*_*s*_). The effect of increasing the overall kill rate (i.e. broadening the range for which high rate of adaptation can be attained) is limited at this higher overall growth rate, such that this phenomenon does not become completely independent of the differences in growth rates. Growth rate (*k*_*s*_) was fixed at 0.05 hr^−1^. (**b**) Differential growth rates give rise to particular regions with distinct high rate of adaptation. In regions where the preferential growth and killing of the subpopulation coincides, the area of this region is diminished (in comparison with Fig. 3b). Killing rate (*α*_*s*_) was fixed at 0.04 hr^−1^.

Thus far, we have analyzed independently the effects of overall growth rate (*k*_*s*_) and overall kill rate (*α*_*s*_). To gain an understanding of the combinatorial effects of these two parameters in relation to differential growth and kill rates, we ran our simulations again with higher overall growth/kill rates. In comparison to Fig. 3a, we again observed the broadening of the region of high rate of adaptation with increased overall killing rate (Fig. 4a and Supplementary Fig. S5a). However, at the higher basal overall growth rate, the spread of this area is now considerably dampened – specifically in regions where the same subpopulation has both a preferential fitness advantage and a preferential killing by drug (i.e. upper half of the heatmap) (Fig. 4a in comparison with Fig. 3a). We obtained similar conclusions when varying overall growth rate at a higher basal overall kill rate (region I in Fig. 3b in comparison with Fig. 4b and Supplementary Fig. S5b), or when we examined the final tumor size (Supplementary Figs S6 and S7 in comparison with Supplementary Figs S2 and S3). On the other hand, in regions where the subpopulation with preferential fitness advantage is not the same subpopulation with preferential killing (i.e. lower half of the heatmap), we observed enhanced spread of areas with high rate of adaptation. Thus, the overall growth and kill rates are competing factors in controlling the range of differential growth and kill rates where high rate of adaptation is attainable, and this is contingent on the directionality of preferential growth and kill.

### Experimental differential selection demonstrating its effect on long-term resistance potential

**Figure 5:**
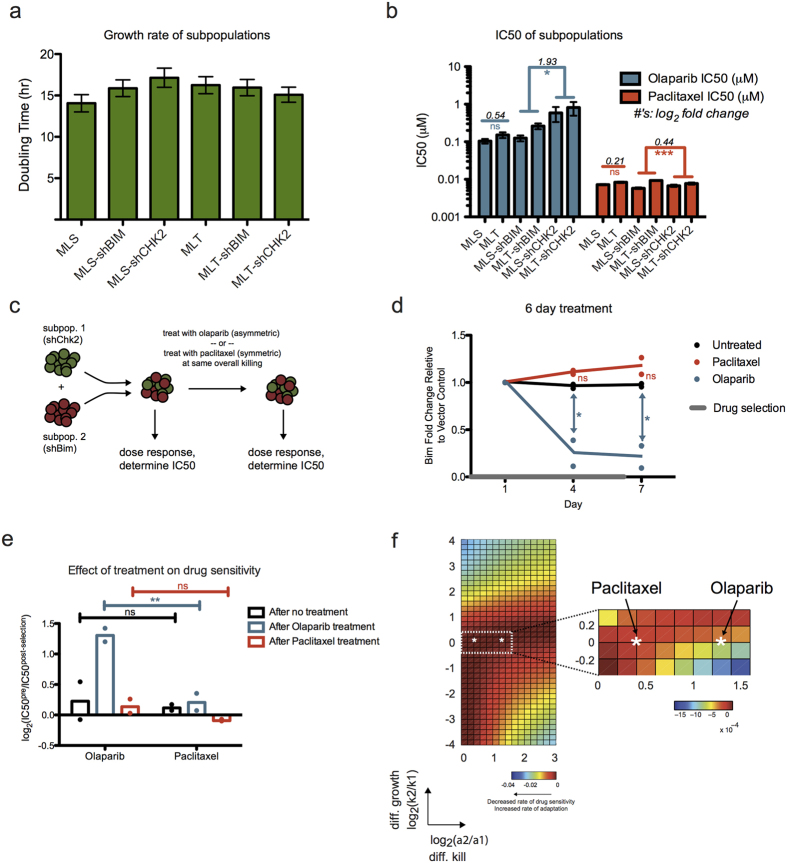
Experimental validation of ‘symmetric’ and ‘asymmetric’ treatments on Eμ-myc tumor cells. (**a**) Parental murine Eμ-myc; p19^Arf−/−^ cell line was transduced with different shRNA hairpins. The bar plot shows the measured net growth rate of the individual cell lines. Data were compiled from three replicates at seven time points. Data is shown as mean ± SEM. No significant difference was observed between any of the growth rates (one-way ANOVA). (**b**) Corresponding IC50s (derived from a 12-point dose response curve, with error bars indicating SEM from model fit) for the individual cell lines upon treatment with olaparib or paclitaxel. For vector controls, IC50 fold change of MLT/MLS is shown. For shChk2/shBim, the geometric mean in magnitude of IC50 fold change between shChk2 and shBim is shown. ns, not significant, *P < 0.05, **P < 0.01, ***P < 0.001 (ANOVA with Bonferroni post-test. p-values for shBim and shChk2 were combined with Fisher’s Method). (**c**) Schematic of the drug selection on an admixed tumor population. Cell lines with shChk2 or shBim were mixed in a 1:1 ratio. IC50s of the cell populations in response to different drugs were determined from dose responses performed pre- and post- drug selection. (**d**) Enrichment/depletion of the shBim fraction relative to the empty vector control and normalized to day 1 shBim fraction over the course of drug selection. Olaparib treatment led to significant depletion of the shBim subpopulation. Data were compiled from two independent experiments. Averages shown as lines, individual replicate values shown as points. ns, not significant, *P < 0.05 (one-way ANOVA with Bonferroni post-test). (**e**) Comparison of shBim/shChk2 populations’ change in drug resistance after symmetric (paclitaxel) or asymmetric (olaparib) treatment. This is calculated here as log_2_ fold change in IC50 between pre- and post- treatment. Data were compiled from two independent experiments. Averages shown as bars, individual replicate values shown as points. ns, not significant, **P < 0.01 (two-way ANOVA with Bonferroni post-test). (**f**) Parameterization of the mathematical model with experimentally derived parameter set (see Table S2) denoted by the white asterisk in the parameter space. Heatmap with the closest match to experimentally derived parameter values was based on *α*_*s*_ = 0.01 hr^−1^ and *k*_*s*_ = 0.05 hr^−1^. Per both computational modeling and experimental results, the asymmetric olaparib treatment led to higher rate of adaptation.

To experimentally validate the results of our model, we used a preclinical murine model of Burkitt’s lymphoma^37^,^38^. The cell line was derived from a *Eμ-myc; p19*^*Arf−/−*^ transgenic mouse, and was additionally transduced with different shRNA hairpins, knocking down genes in the DNA damage pathway and Bcl-2 family members^33^,^39^. Each sorted population, with shRNA targeting a gene of interest, represents a distinct subpopulation. A heterogeneous tumor can thus be modeled by using admixed combinations of these subpopulations. Empty vectors were used as negative controls. We measured the net growth rate in the absence of drug and dose response in the presence of a number of chemotherapeutics for each individual subpopulation (Supplementary Fig. S8). From these, we chose a pair of subpopulations with the same/differential sensitivity to distinct drugs. We observed no statistically significant differences between growth rates of the chosen cell lines: shBim and shChk2 (Fig. 5a). Both shBim and shChk2 conferred similar sensitivity to paclitaxel treatment, a microtubule poison, while shBim conferred significantly more sensitivity to a PARP inhibitor, olaparib, than shChk2 (Fig. 5b). Because of the large difference in IC50s for shBim and shChk2 under olaparib treatment, this drug is an example of asymmetric treatment. Conversely, the modest difference in IC50s for these two subpopulations under paclitaxel shows that this is a case of symmetric treatment. As expected, we observed no significant differences in IC50s for our MLT and MLS vector controls for both drugs.

Using an admixed population of shBim/shChk2 on MLS or MLT vectors in equal proportions, we exposed the heterogeneous tumor population to olaparib or paclitaxel at a dose that killed 50% of the overall population. Both before and after drug selection, we performed dose responses to measure the efficacy of olaparib and paclitaxel on the original and post-selected populations (Fig. 5c). Under little selective pressure and nearly symmetric treatment with paclitaxel, the admixed tumor populations maintained their subpopulation proportions. We observed no significant difference in shBim percentage, relative to the MLS/MLT control population, between untreated and paclitaxel-treated populations over 7 days of treatment (Fig. 5d). We also observed no significant changes in drug sensitivity in the untreated and paclitaxel treated populations post-selection (Fig. 5e). In contrast, upon asymmetric treatment with olaparib, shBim depletion was observed relative to the vector control, showing significant difference in shBim fold change compared to untreated populations by 4 and 7 days of treatment (Fig. 5d). Furthermore, the resulting post-selection population showed significant increase in resistance to subsequent olaparib treatment (Fig. 5e).

Using the experimentally determined growth rates and killing rates (Supplementary Table S2), we can further parameterize our mathematical model to visualize where our experimentally validated parameter set lies using the same schematic of differential growth versus differential kill that we have introduced earlier. Here, we observed that the asymmetric drug olaparib, with a higher differential selective pressure, exhibited a higher rate of adaptation (Fig. 5f). Notably, the range in rate of adaptation from our computational model was quite broad, and even the modest difference, per prediction by the model, between these two drugs was sufficiently resolved experimentally. Taken together, knowledge of tumor subpopulation growth rates and kill rates can enable predictions on how drug treatment affects rate of adaptation. While drugs with intrinsic asymmetric selective pressure on individual subpopulations can achieve equally effective initial response compared to symmetric regimens, they can amplify rate of adaptation and eventual resistance acquisition.

## Discussion

Chemotherapy regimens have been traditionally developed empirically with the goal of maximizing overall tumor kill while maintaining a tolerable level of toxicity. This empirical approach and such rationales have been the major cornerstone for many of the frontline combination therapies (e.g. Hyper-CVAD, CHOP, etc) used even to this day. However, although this ‘hit-hard, hit-early’ approach has led to many success stories, drug resistance remains a major obstacle towards achieving a cure. In addition, recent genome sequencing of patients both longitudinally and spatially in different regions of the primary/metastatic tumor have brought greater focus to the extent of intratumoral heterogeneity in patients^40^. This presents a major challenge for therapeutic strategies in minimizing resistance, as we often observe the outgrowth of minor subpopulations to dominance at relapse^41^. This is further complicated by the complexity of potential differences in fitness for each subpopulation, both in terms of their growth rates and their responses to drugs.

Here, using ordinary differential equations modeling in a tractable model, we have systemically explored how different contexts (i.e. subpopulation size, growth and kill rates) affect outcome (using metrics such as final tumor size and drug sensitivity). We observed that the initial tumor response is often not the best predictor for eventual prognosis. We specifically highlighted this through comparing regimens where the initial overall kill (i.e. selective pressure imposed by the drug) is the same, but due to differential selective pressure on the subpopulations, these regimens can result in very different therapeutic outcomes. A systematic interrogation through parameter sampling further enabled us to rationalize this effect in the context of different magnitudes of initial subpopulation proportions, and growth and kill rates of overall and individual subpopulations. We further illustrated this using a preclinical murine model of Burkitt’s lymphoma (*Eμ-myc; p19*^*Arf−/−*^), where treatment of an admixed heterogeneous tumor population with olaparib (a case of a differential selective pressure regimen) led to much greater resistance potential than paclitaxel (a case of an equal selective pressure regimen).

Furthermore, we observed the intrinsic tradeoff in maximizing overall kill (and thus greater initial reduction in tumor size) and greater resistance potential. In other words, while we can modulate parameters by choosing drugs that result in increased overall tumor killing, such strategies often come at the expense of drug sensitivity after repetitive treatments. This concept has been explored in antibiotic combination studies, where highly synergistic antibiotic drug combinations (i.e. greater selective pressure imposed on the population) led to a much greater rate of adaptation^9^,^42^. Therefore, consideration of the impact of overall and individual selective pressures on the rate of resistance acquisition is equally as critical as the initial tumor response.

Our model was based on the assumption that selection dominates over *de novo* mutation and genetic drift as the main driver of resistance in regimes where there exists a minor, but substantially sized, population of resistant cells. There are other clinically relevant contexts where drift and *de novo* mutations cannot be ignored. This includes, for example, when the resistant subpopulation is not present prior to treatment and/or in very small numbers, such that mutations and drift can vastly influence the dynamics and timing of clonal occurrence, and the overall time to relapse. Several studies of tumor clonal dynamics have used various stochastic methods to model *de novo* mutation and/or genetic drift in cancer initiation/progression^13^,^16^,^22^,^23^ and response to drug treatment^36^. Additional factors such as different growth models, clonal interference, and other subpopulation interactions (and with its environment) would undoubtedly further complicate our interpretations. Nevertheless, this study offers an analysis on the tradeoffs in short-term and long-term drug response from the perspective of tumor dynamics driven by selection.

## Computational/Mathematical Methods

### Mathematical model

A heterogeneous tumor was modeled as two subpopulations (*P*_1_, 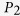), with distinctive growth rates (*k*_1_, *k*_2_) and drug killing rates (*α*_1_, *α*_2_). The kinetics of the two subpopulations were modeled using ODE equations for exponential growth, shown below.


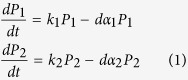


Drug treatment (*d*) was described using a Heaviside step function. Given the constraints of same initial overall tumor growth and tumor reduction (for the first treatment), the system can be completely defined by 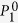, 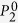, *k*_1_, *α*_1_, *k*_*s*_, *α*_*s*_. The parameters *k*_*s*_ and *α*_*s*_ define the overall growth and kill rate, respectively. Given a set of values for these parameters and applied constraints, we can subsequently derive the values for *k*_2_ and *α*_2_.

### Boundary and constraints prior to treatment

During the initial untreated growth phase of the tumor, in order satisfy the constraint of the same initial tumor burden prior to treatment (time point t), the *k*_1_ and *k*_2_ values are such that the total tumor size is equivalent to a single overall growth rate, *k*_*s*_.


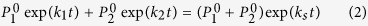


where 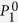 and 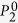 are initial subpopulation sizes.

Solving for *k*_2_


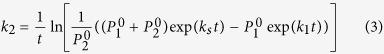


The maximum *k*_1_ value such that *k*_2_ is defined and nonnegative,


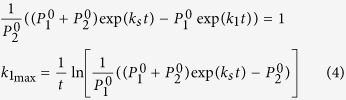


**Lemma 1.1:** 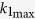 is always greater than *k*_*s*_, and as such any *k*_2_ values equal to or less than *k*_*s*_ are always defined.

**Proof of Lemma 1.1:**

As defined above, the expression 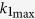 > *k*_*s*_ can be expanded to


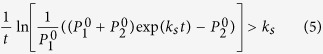


To prove Lemma 1.1 is to demonstrate this inequality is true. The above expression (5) can be reduced to


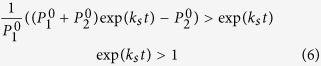


Since *k*_*s*_ is always a positive value, the expression exp(*k*_*s*_*t*) is always greater than 1.

### Boundary and constraints following first round of drug treatment

Per our constraint, after the first dose, the differential killing of the subpopulations should result in equivalent overall tumor burden reduction as per overall growth rate (*k*_*s*_) and kill rate (*α*_*s*_), such that,


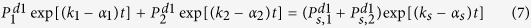


Solving for *α*_2_


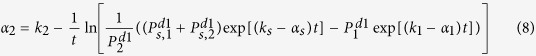


where 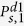 and 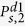 are subpopulations *P*_1_ and *P*_2_ under symmetric growth (*k*_*s*_) at time point *d1*.

**Lemma 1.2:** The bound for *α*_1_ is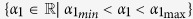 and for *α*_2_ is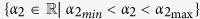, where


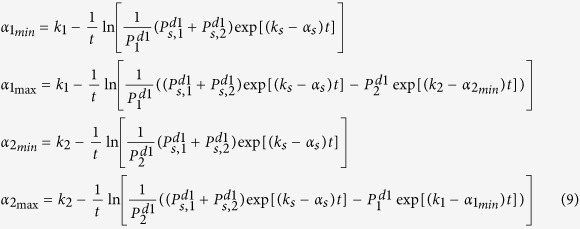


**Proof of Lemma 1.2:**

The minimum *α*_1_ value such that *α*_2_ is defined requires


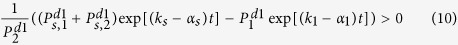


Therefore,


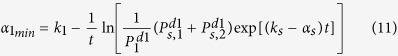


The value *α*_2_ is maximum when the logarithm term in the *α*_2_ expression is minimized, which occurs when 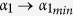. This means,


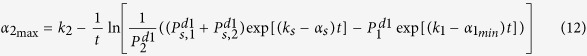


An additional constraint is to ensure that *α*_2_ is not negative. Therefore, setting *α*_2_ equal to zero and rearranging the terms in (8),


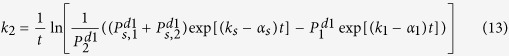


Solving for *α*_1_,


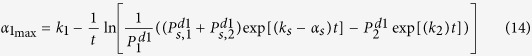


Similarly, solving for *α*_1_ from equation (7),


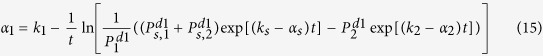


which means the minimum *α*_2_ for which *α*_1_ is defined is,


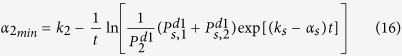


And the maximum value for *α*_1_ is,


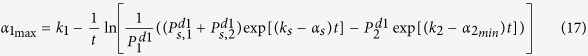


It is clear that the expression for 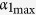 above is a tighter bound (vis-à-vis equation (14)) because of the extra 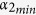 term. These bounds for 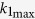 (equation (4)) and 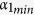, 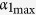, 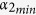, and 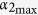 (Lemma 1.2 equations in (9)) were used when we performed parameter sampling (see next section below).

### Parameter sampling and simulations

We used a Latin hypercube sampling method to explore the global parameter space. Specifically, we performed a parameter sweep of 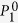, 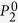, *k*_*s*_, *α*_*s*_, log2[*α*_2_/*α*_1_], *k*_2_/*k*_1_. Here we sampled the ratios of the killing and growth rates instead of *α*_1_ or *k*_1_ to ensure uniform sampling in this transformed space – as this is the final parameter space in which we performed our subsequent analyses. From the sampled ratios, we derived the corresponding *α*_1_ via non-linear regression fit of log2[*α*_2_/*α*_1_] versus *α*_1_ to an exponential function. Similar fitting was performed to derive *k*_1_. For each given parameter set {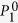, 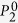, *k*_*s*_, *α*_*s*_, *k*_1_, *α*_1_}, we calculated the corresponding *k*_2_ and *α*_2_ values according to equations (3) and (8). We only kept values that are within bounds, as described in our constraint analyses. ODE simulation was then performed using Matlab ODE solver ode45 with a relative error tolerance of 1e–8 and an absolute error tolerance of [1e–8, 1e–8].

## Experimental Methods

### Cell culture and chemicals

Murine Eμ-Myc; p19^Arf −/−^ B-cell lymphomas were cultured in B-cell medium [45% Dulbecco’s Modified Eagle Medium (DMEM), 45% Iscove’s Modified Dulbecco’s Medium (IMDM), 10% FBS, supplemented with 2 mmol/L L-glutamine, and 5 μmol/L β-mercaptoethanol]. The cell line was tested and shown to be free of Mycoplasma using both PCR-based (American Type Culture Collection) and biochemical-based (Lonza) methods. Paclitaxel was obtained from LC Laboratories and olaparib was a gift from Peter Ghoroghchian.

### shRNA constructs and transfection/transduction

All shRNAs were expressed in either MSCV–LTR–MIR30–SV40–GFP (MLS)^43^ or MSCV–LTR–MIR30–PGK–Tomato (MLT) retroviral vectors and were previously validated for knockdown^39^. Transfection and transduction were performed as previously described^33^. Infected cells were subsequently sorted using a cell sorter.

#### *In vitro* assays

Proliferation assay was performed to determine the net growth rate of each sorted shRNA-containing population. On day 0, 10,000 cells were seeded per well in a 96 well plate and incubated for up to 72 hrs. Cell number was counted every 12 hrs using a cell analyzer FACS LSR, FACSCanto, or LSRFortessa (BD Biosciences). For dose response experiments, the kill rates were assessed for individual populations by measuring the IC50 at 48 hours. The IC50 value was determined via fitting data to the following dose response equation,


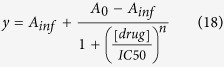


where y is the % live cells, *A*_*inf*_ is the % live cells at the highest drug concentration, *A*_0_ is the % live cells at 0 drug concentration, n is the hill coefficient, IC50 is the drug concentration where the % live cells is reduced by half, and [drug] is the concentration of the drug.

Killing rate *α* was calculated using the following equation.


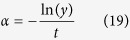


For drug selection experiments, indicated shRNA-containing populations were mixed at a 50:50 ratio and confirmed using a cell analyzer. The admixed tumor population was treated with indicated drug at IC50 concentration – a dose needed to achieve 50% cell death at 48 hours in the individual populations. Cells were cultured in drug for six days and were split as needed. Cell death was assessed with propidium iodide incorporation. After four days, the percentages of GFP+ and Tomato+ cells were analyzed on a cell analyzer. Experiments were performed with indicated shRNAs in both MLS and MLT vectors to rule out any vector-specific effects.

### Experimentally Derived Model Parameters


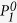
 and 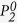 values were determined based on the final ratio of the two populations after several rounds of mixing, averaged between the two replicates (ratios experimentally determined using FACS). *k*_*1*_ and *k*_*2*_ values are the experimentally determined shChk2 and shBim growth rates in the absence of drug, averaged between the two vectors. *k_s_* was solved for based on the determined values for 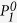, 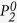, *k_1_*, and *k_2_*, following the constraint equation for symmetric initial tumor burden before treatment (2). *α_1_* and *α_2_* values for each drug were derived from individual dose response experiments, averaged between the two vectors, and calculated based on equations (18) and (19). *α_s_* was calculated using equation (19), where the percent live cells (y) was 50%, based on the experimental design criteria that the admixed tumor population would be treated at a dose achieving 50% cell death at 48 hours.

### Statistical Analyses

Statistical analyses were performed using Prism v5 (GraphPad). Two-tailed Student *t* tests, ANOVA and two-way ANOVA with Bonferroni’s post hoc tests, and Fisher’s method were performed as indicated. Error bars shown are mean ± SEM.

## Additional Information

**How to cite this article**: Sun, D. *et al.* Differential selective pressure alters rate of drug resistance acquisition in heterogeneous tumor populations. *Sci. Rep.* **6**, 36198; doi: 10.1038/srep36198 (2016).

**Publisher’s note**: Springer Nature remains neutral with regard to jurisdictional claims in published maps and institutional affiliations.

## Supplementary Material

Supplementary Information
