# Supplementary material for: Differential selective pressure alters rate of drug resistance acquisition in heterogeneous tumor populations
Source: Sci Rep. 2016 Nov 7;6:36198. doi: 10.1038/srep36198 (PMC5098152; doi:10.1038/srep36198)
Supplement: Supplementary Information [file srep36198-s1.pdf]

Supplemental Information for *Differential selective pressure alters rate of drug resistance acquisition in heterogeneous tumor populations*

Daphne Sun[1], Simona Dalin[2,3], Michael T. Hemann[2,3], Douglas A. Lauffenburger[1,2,3,*], Boyang Zhao[1,3,4,*]

[1]Department of Biological Engineering, [2]Department of Biology, [3]The David H. Koch Institute for Integrative Cancer Research, [4]Computational and Systems Biology Program, Massachusetts Institute of Technology, Cambridge, MA 02139

SUPPLEMENTAL FIGURE LEGENDS

Figure S1. Effects of variations on kinetics parameters on rate of adaptation: auto-scaled heat maps. These are the same heatmaps as in Fig. 3, with auto-scaled coloration to better observe effects in each simulation. Note that the colorbar scaling is different for each heatmap.

Figure S2. Effects of variations of kinetics parameters on final tumor size. Heat maps showing final tumor size, corresponding to simulation results displayed in Fig. 3. (a) Increasing the overall kill rate broadens the region for which the differential kill rates (traversing the abscissa) affects tumor size, but generally decreases the overall final tumor size (see Supplementary Fig. S3a). (b) Increasing the overall growth rate broadens the region of increased tumor size due to differential growth rates (see Supplementary Fig. S3b) and generally increases overall final tumor size. (c) Decreasing the proportion of the more resistant subpopulation 1 in the initial tumor decreases the overall final tumor size, but preserves the general effects of differential growth and kill rates (see Supplementary Fig. S3c).

Figure S3. Effects of variations on kinetics parameters on final tumor size: auto-scaled heat maps. Same heat maps as Supplementary Fig. S2, auto-scaled.

Figure S4. Effects of increasing overall kill rate on final tumor size and rate of adaptation.
(a-b) Comparison of tumor kinetics between treatments with low (subpanel a, α_S= 0.04 hr^{-1}) and high (subpanel b, α_S = 0.1 hr^{-1}) overall killing rates (α_s), under the same differential growth and differential killing on the subpopulations. The black asterisk indicates an instance of a specific parameter set combination used for simulation. Right panel shows the simulation results based on this parameter set –

illustrating the outgrowth of a fitter subpopulation 1 (blue) upon successive drug treatments. (c-d) Tumor kinetics (c) and percent tumor reduction (d) for the same simulation based on the parameter sets indicated by black asterisks in (a) and (b). Rate of adaptation can be extracted as the slope in (d). This highlights an intrinsic tradeoff, where increasing the overall kill rate leads to a lower overall tumor size, but a faster rate of adaptation.

Figure S5. Competing effects of variations on multiple kinetics parameters on rate of adaptation: auto-scaled heat maps. Same heat maps as Fig. 4, auto-scaled.

Figure S6. Competing effects of variations on multiple kinetics parameters on final tumor size. Heat maps showing metric of final tumor size, corresponding to simulation results displayed in Fig. 4. (a) Increasing overall kill rate increases final tumor size and broadens the range for higher final tumor size due to increased differential kill rates (see Supplementary Fig. S7a). However, this effect is limited by the higher basal overall growth rate (k_S fixed at 0.05 hr^{-1}). (b) Increasing overall growth rate at higher basal overall kill rate (α_S fixed at 0.04 hr^{-1}) limits the effect of differential growth rates on tumor size and results in decreased final tumor size (compared to Supplementary Fig. S2b and Supplementary Fig. S3b**).**

Figure S7. Competing effects of variations on multiple kinetics parameters on final tumor size: auto-scaled heat maps. Same heat maps as Supplementary Fig. S6, auto-scaled.

Figure S8. Initial screen of knockdown populations and drugs. (a) Parental murine Eμ-myc; $p19^{Arf-/-}$ cell line was transduced with different shRNA hairpins. The bar plot shows the measured net growth rate of the individual cell lines. Data were compiled from three replicates at seven time points. Data is shown as mean ± SEM. No significant difference was observed between any of the growth rates (one-way ANOVA). (b) Relative difference in IC50s between each pair of cell lines under treatment with 17AAG, dacarbazine, doxorubicin, etopiside, olaparib, or paclitaxel, shown as the absolute value of the $\log_2$(MLS-hairpin/MLT-hairpin). (Raw IC50s derived from a 12-point dose response curve). The drug/hairpin combinations chosen for further investigation are indicated with black boxes.

Figure S1

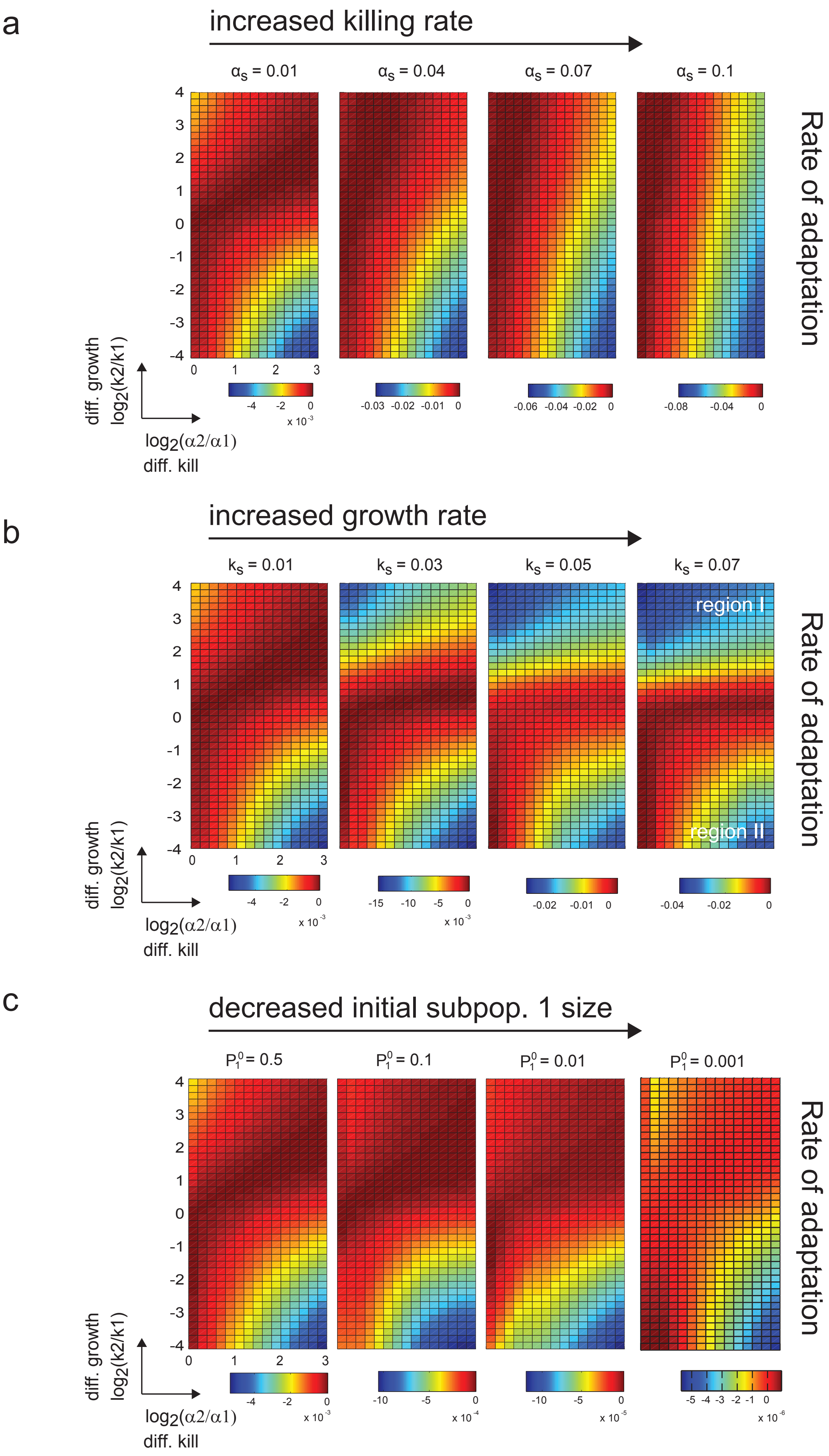
a
increased killing rate
αs = 0.01
αs = 0.04
αs = 0.07
αs = 0.1
Rate of adaptation
diff. growth
log2(k2/k1)
log2(α2/α1)
diff. kill
x 10-3
b
increased growth rate
ks = 0.01
ks = 0.03
ks = 0.05
ks = 0.07
region I
region II
Rate of adaptation
c
decreased initial subpop. 1 size
P10 = 0.5
P10 = 0.1
P10 = 0.01
P10 = 0.001
Rate of adaptation

Figure S2

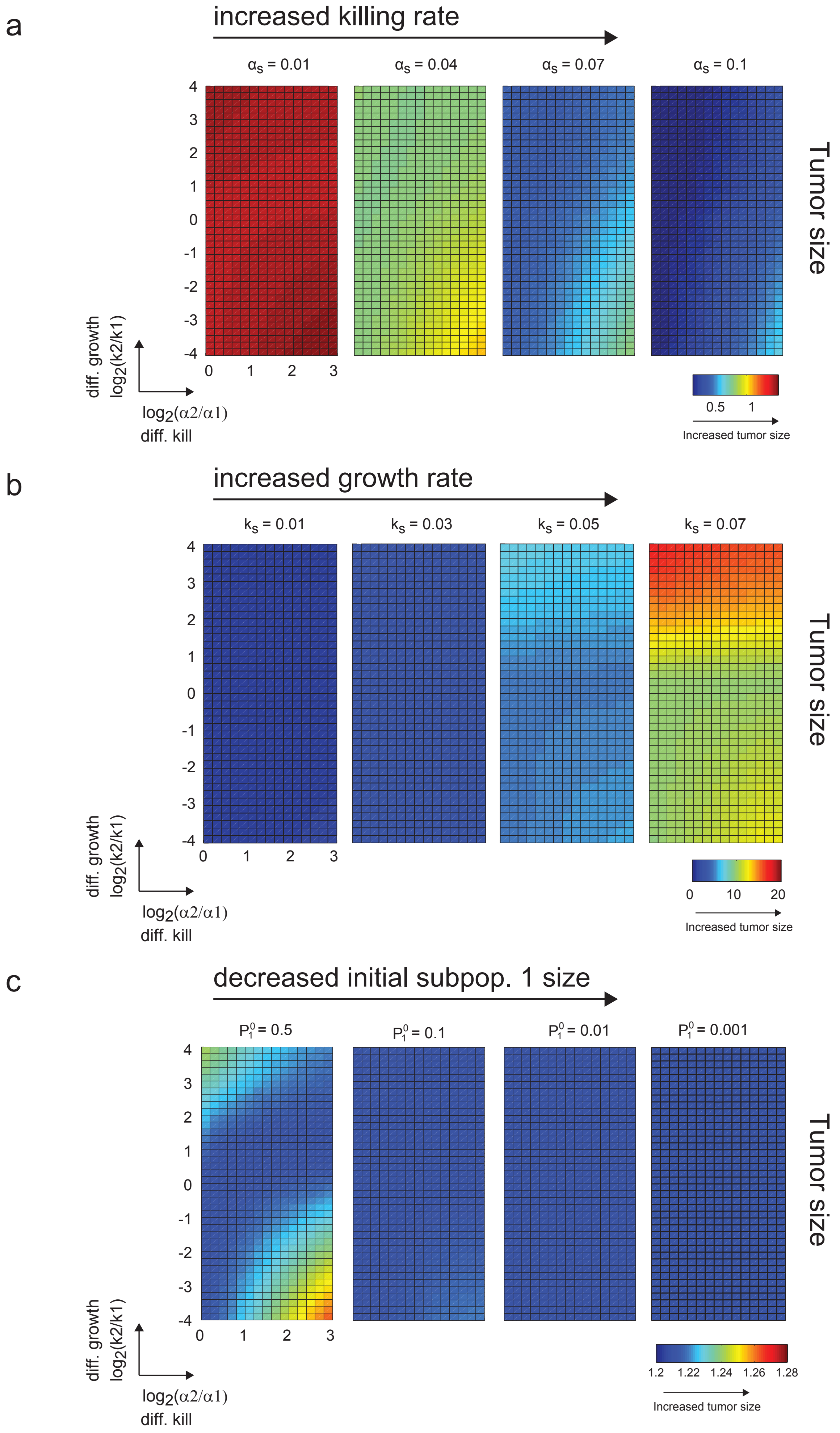

a
increased killing rate
αs = 0.01
αs = 0.04
αs = 0.07
αs = 0.1
4
3
2
1
0
-1
-2
-3
-4
0
1
2
3
Tumor size
diff. growth
log2(k2/k1)
log2(α2/α1)
diff. kill
0.5
1
Increased tumor size
b
increased growth rate
ks = 0.01
ks = 0.03
ks = 0.05
ks = 0.07
4
3
2
1
0
-1
-2
-3
-4
0
1
2
3
Tumor size
diff. growth
log2(k2/k1)
log2(α2/α1)
diff. kill
0
10
20
Increased tumor size
c
decreased initial subpop. 1 size
P10 = 0.5
P10 = 0.1
P10 = 0.01
P10 = 0.001
4
3
2
1
0
-1
-2
-3
-4
0
1
2
3
Tumor size
diff. growth
log2(k2/k1)
log2(α2/α1)
diff. kill
1.2
1.22
1.24
1.26
1.28
Increased tumor size

Figure S3

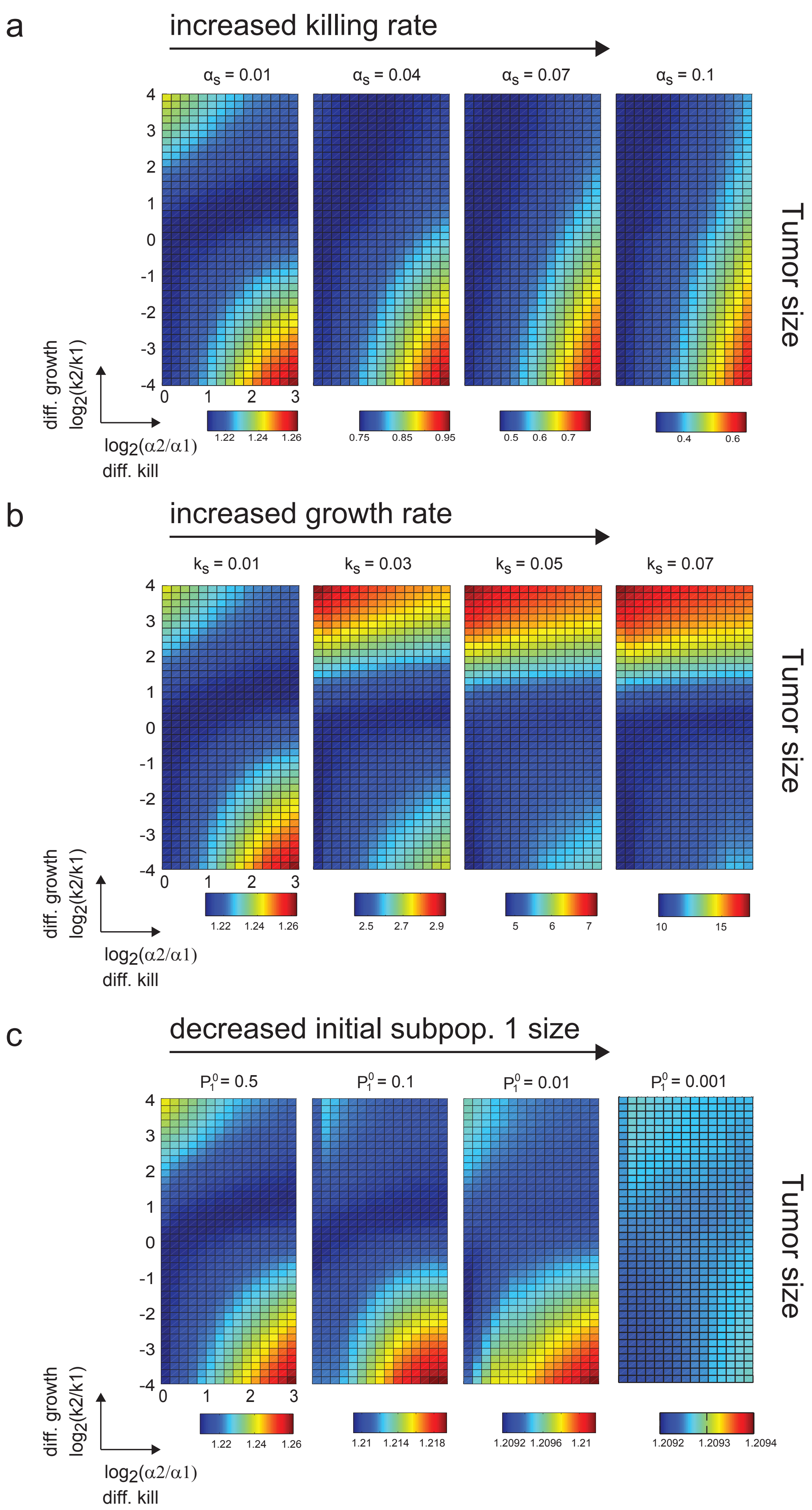
a
increased killing rate
αs = 0.01
αs = 0.04
αs = 0.07
αs = 0.1
4
3
2
1
0
-1
-2
-3
-4
0
1
2
3
1.22 1.24 1.26
0.75 0.85 0.95
0.5 0.6 0.7
0.4 0.6
Tumor size
diff. growth
log2(k2/k1)
log2(α2/α1)
diff. kill
b
increased growth rate
ks = 0.01
ks = 0.03
ks = 0.05
ks = 0.07
1.22 1.24 1.26
2.5 2.7 2.9
5 6 7
10 15
Tumor size
c
decreased initial subpop. 1 size
P1^0 = 0.5
P1^0 = 0.1
P1^0 = 0.01
P1^0 = 0.001
1.22 1.24 1.26
1.21 1.214 1.218
1.2092 1.2096 1.21
1.2092 1.2093 1.2094
Tumor size

Figure S4

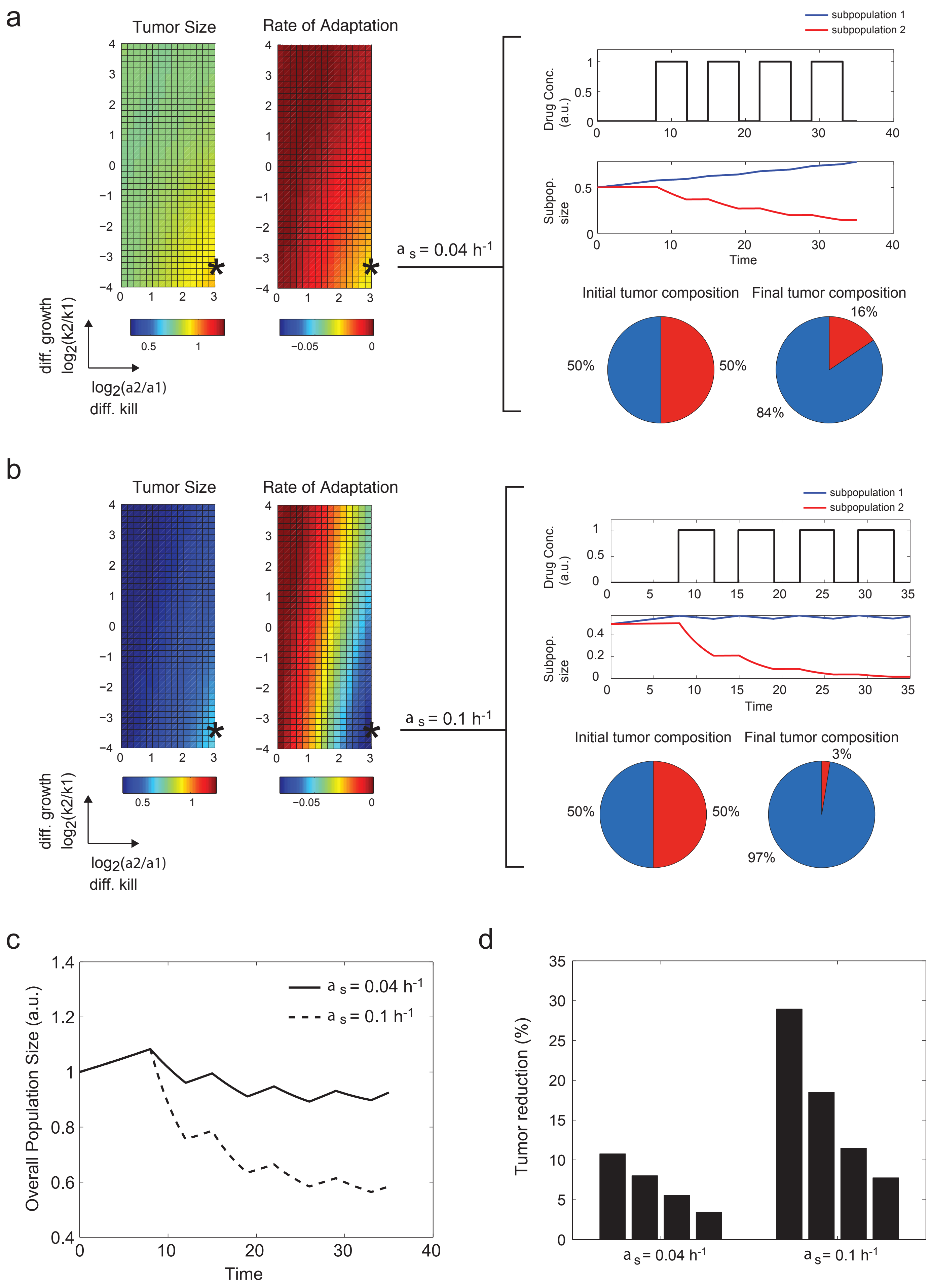

a
Tumor Size
Rate of Adaptation
diff. growth
log2(k2/k1)
log2(a2/a1)
diff. kill
a s = 0.04 h-1
subpopulation 1
subpopulation 2
Drug Conc.
(a.u.)
Subpop.
size
Time
Initial tumor composition
Final tumor composition
50%
50%
16%
84%
b
Tumor Size
Rate of Adaptation
diff. growth
log2(k2/k1)
log2(a2/a1)
diff. kill
a s = 0.1 h-1
subpopulation 1
subpopulation 2
Drug Conc.
(a.u.)
Subpop.
size
Time
Initial tumor composition
Final tumor composition
50%
50%
3%
97%
c
Overall Population Size (a.u.)
Time
a s = 0.04 h-1
a s = 0.1 h-1
d
Tumor reduction (%)
a s= 0.04 h-1
a s= 0.1 h-1

Figure S5

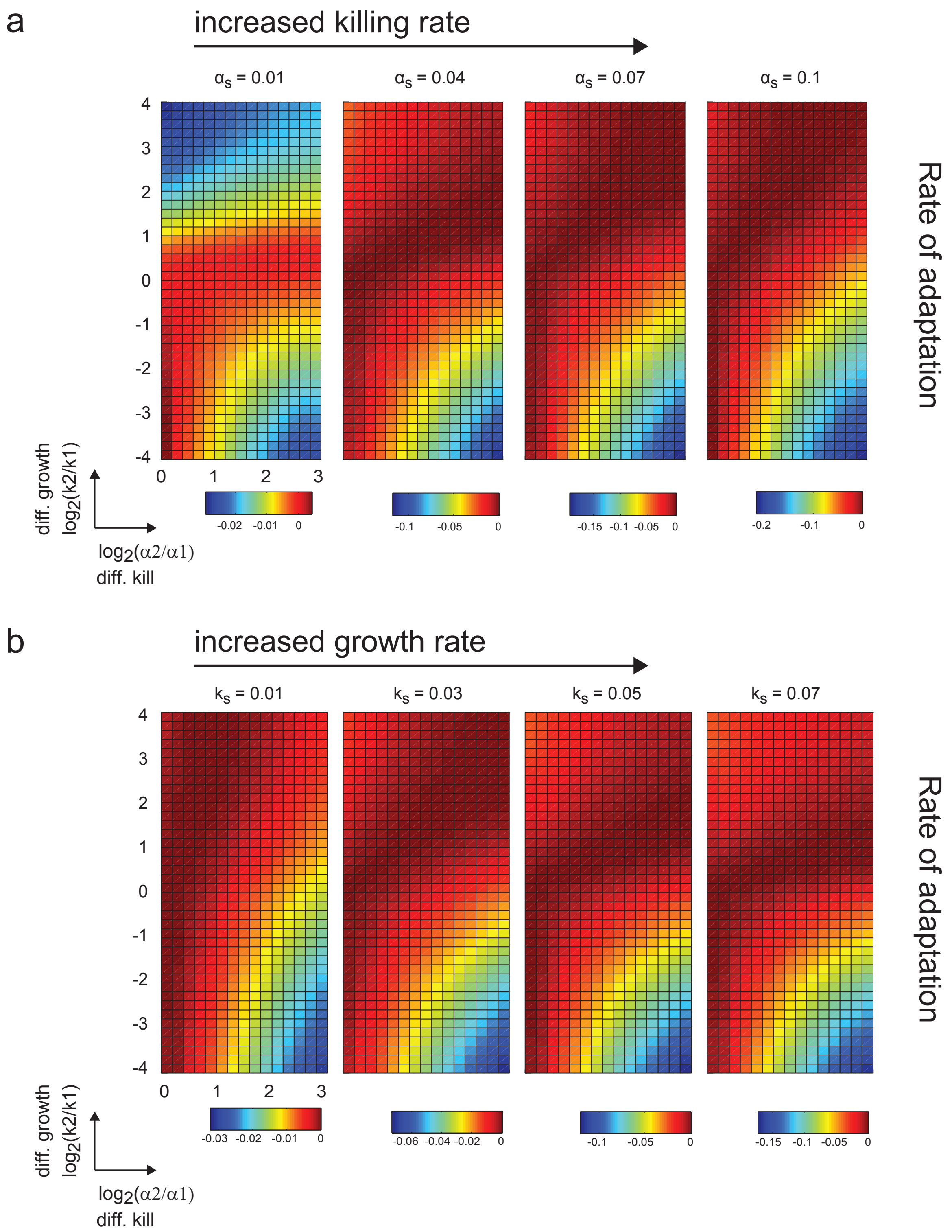
a
increased killing rate
α_s = 0.01
α_s = 0.04
α_s = 0.07
α_s = 0.1
4
3
2
1
0
-1
-2
-3
-4
0
1
2
3
-0.02 -0.01 0
-0.1 -0.05 0
-0.15 -0.1 -0.05 0
-0.2 -0.1 0
Rate of adaptation
diff. growth
log2(k2/k1)
log2(α2/α1)
diff. kill
b
increased growth rate
k_s = 0.01
k_s = 0.03
k_s = 0.05
k_s = 0.07
4
3
2
1
0
-1
-2
-3
-4
0
1
2
3
-0.03 -0.02 -0.01 0
-0.06 -0.04 -0.02 0
-0.1 -0.05 0
-0.15 -0.1 -0.05 0
Rate of adaptation
diff. growth
log2(k2/k1)
log2(α2/α1)
diff. kill

Figure S6

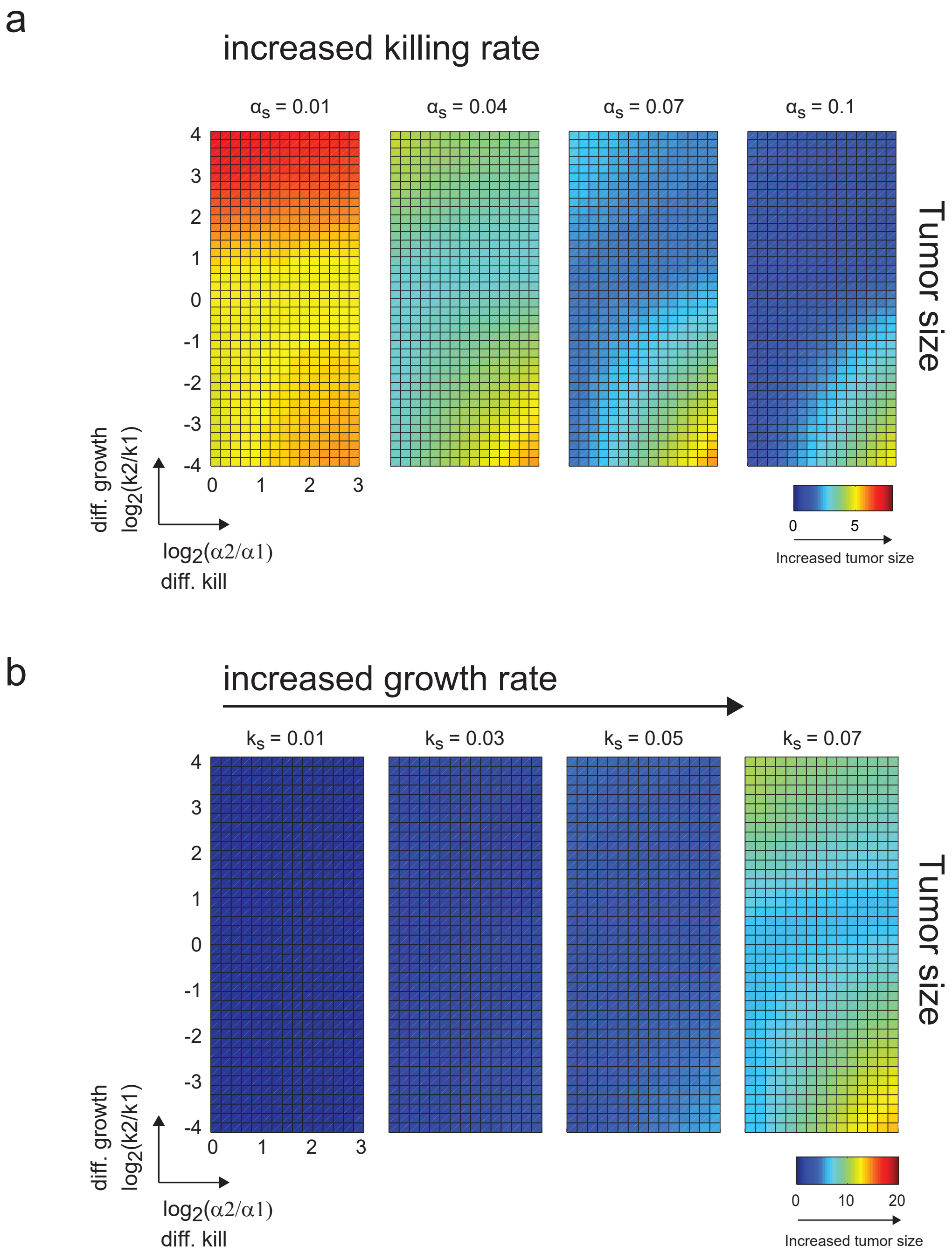
a
increased killing rate
α_s = 0.01
α_s = 0.04
α_s = 0.07
α_s = 0.1
4
3
2
1
0
-1
-2
-3
-4
0
1
2
3
Tumor size
diff. growth
$\log_2(k2/k1)$
$\log_2(\alpha 2/\alpha 1)$
diff. kill
0
5
Increased tumor size
b
increased growth rate
k_s = 0.01
k_s = 0.03
k_s = 0.05
k_s = 0.07
4
3
2
1
0
-1
-2
-3
-4
0
1
2
3
Tumor size
diff. growth
$\log_2(k2/k1)$
$\log_2(\alpha 2/\alpha 1)$
diff. kill
0
10
20
Increased tumor size

Figure S7

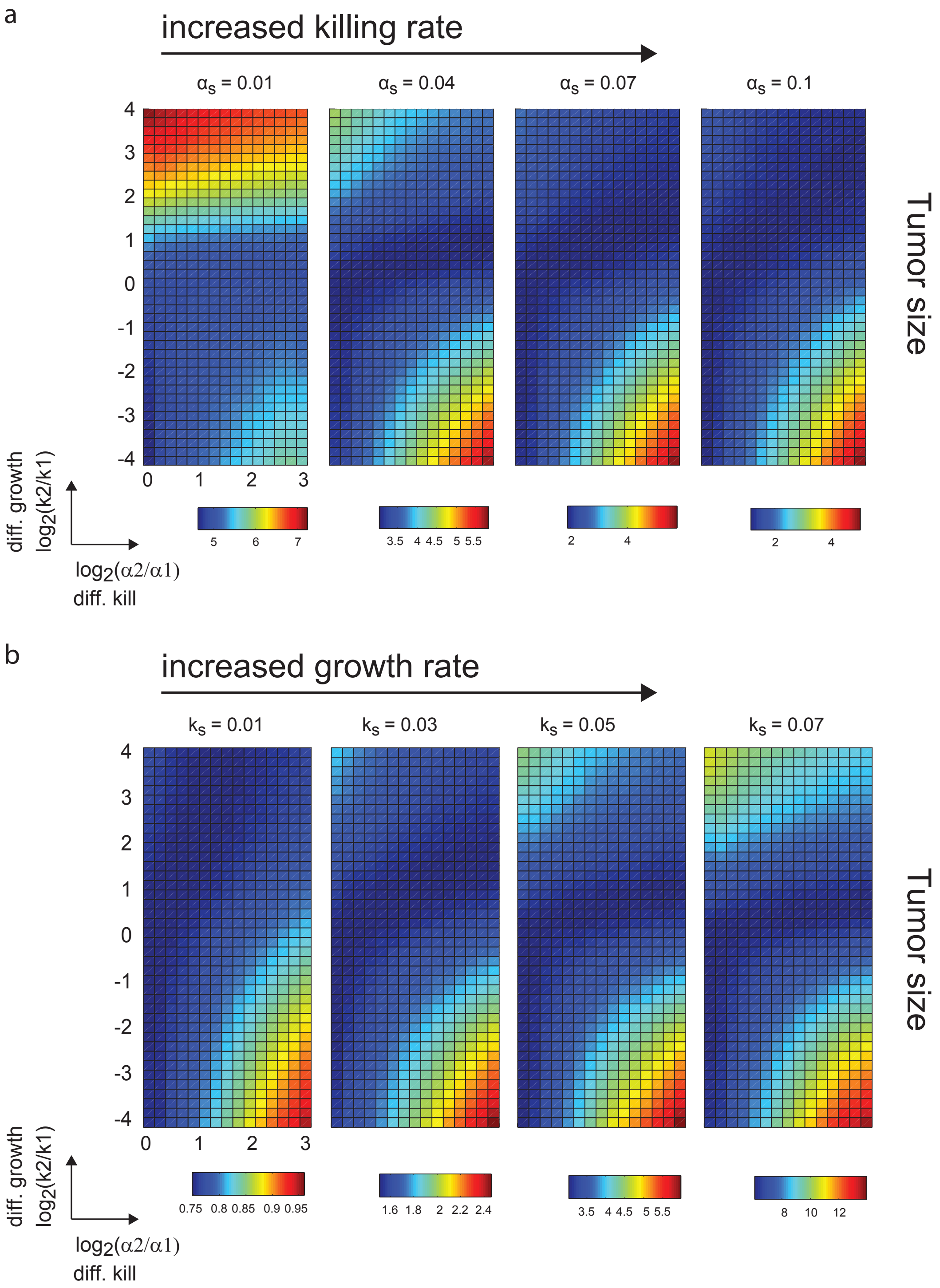
a
increased killing rate
α_s = 0.01
α_s = 0.04
α_s = 0.07
α_s = 0.1
Tumor size
4 3 2 1 0 -1 -2 -3 -4
0 1 2 3
5 6 7
3.5 4 4.5 5 5.5
2 4
2 4
diff. growth
$\log_2(k2/k1)$
$\log_2(\alpha2/\alpha1)$
diff. kill
b
increased growth rate
k_s = 0.01
k_s = 0.03
k_s = 0.05
k_s = 0.07
Tumor size
4 3 2 1 0 -1 -2 -3 -4
0 1 2 3
0.75 0.8 0.85 0.9 0.95
1.6 1.8 2 2.2 2.4
3.5 4 4.5 5 5.5
8 10 12
diff. growth
$\log_2(k2/k1)$
$\log_2(\alpha2/\alpha1)$
diff. kill

Figure S8

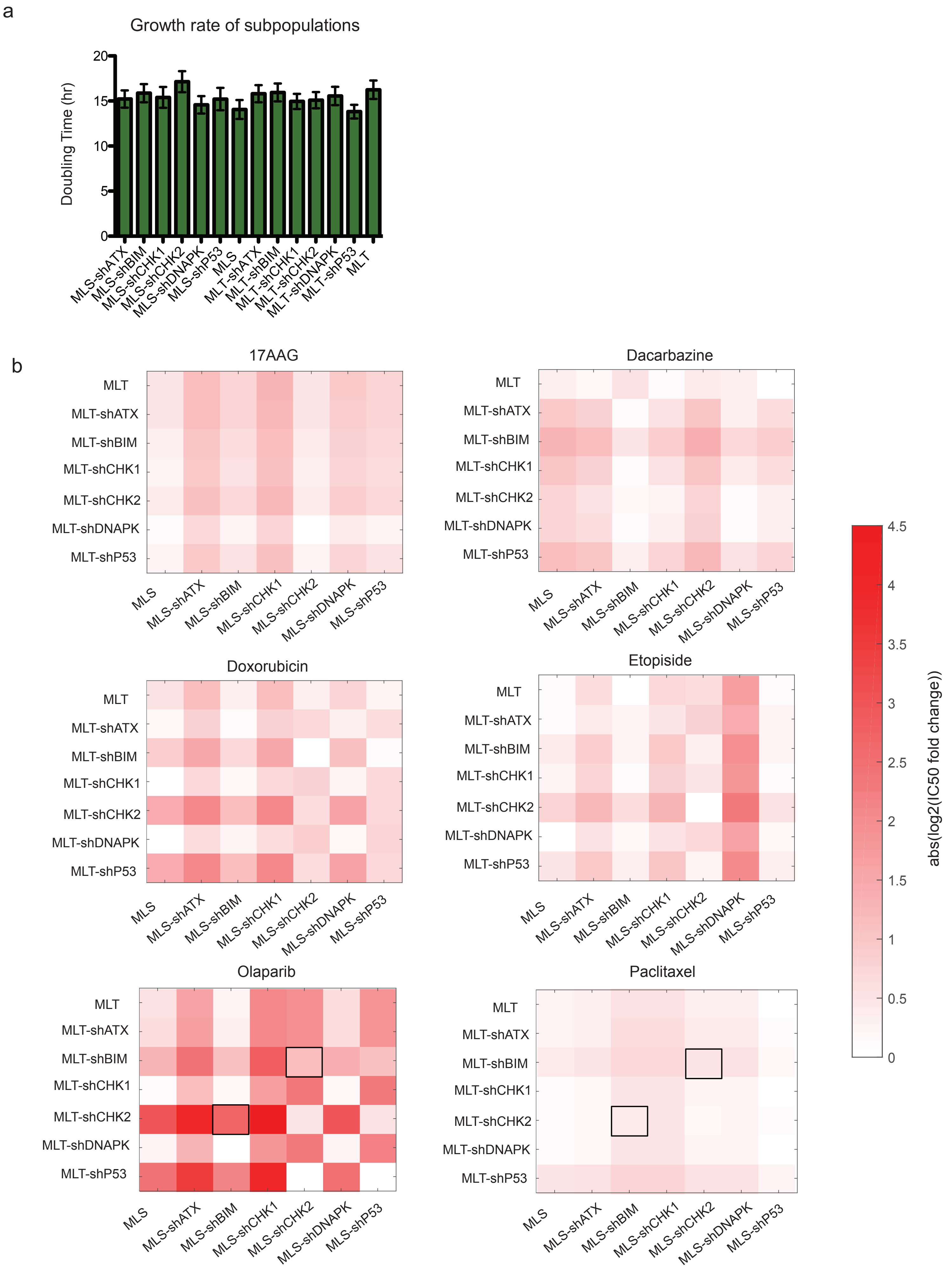
a
Growth rate of subpopulations
Doubling Time (hr)
0
5
10
15
20
MLS-shATX
MLS-shBIM
MLS-shCHK1
MLS-shCHK2
MLS-shDNAPK
MLS-shP53
MLS
MLT-shATX
MLT-shBIM
MLT-shCHK1
MLT-shCHK2
MLT-shDNAPK
MLT-shP53
MLT
b
17AAG
Dacarbazine
Doxorubicin
Etopiside
Olaparib
Paclitaxel
MLT
MLT-shATX
MLT-shBIM
MLT-shCHK1
MLT-shCHK2
MLT-shDNAPK
MLT-shP53
MLS
MLS-shATX
MLS-shBIM
MLS-shCHK1
MLS-shCHK2
MLS-shDNAPK
MLS-shP53
abs(log2(IC50 fold change))
0
0.5
1
1.5
2
2.5
3
3.5
4
4.5

Table S1. Parameter sampling ranges used for simulations

Parameter	Unit	Description	Range sampled
α_s	hr^{-1}	Overall killing rate	0.01 – 0.1
k_s	hr^{-1}	Overall growth rate	0.01 – 0.07
P_1^0	a.u.	Initial subpop. 1 fraction	0.01 – 0.5
P_2^0	a.u.	Initial subpop. 2 fraction	0.5 – 0.99
$\log2[\alpha_2/\alpha_1]$		Log2 subpop. killing ratio	0 – 3
k_2/k_1		Subpop. growth rate ratio	0 – 6

Table S2. Experimentally Derived Parameters

Parameter	Unit	Value under olaparib treatment	Value under paclitaxel treatment
P_1^0 (initial proportion of shChk2)		0.446	0.446
P_2^0 (initial proportion of shBim)		0.541	0.541
k_1 (growth rate of shChk2)	hr^{-1}	0.0432	0.0432
k_2 (growth rate of shBim)	hr^{-1}	0.0436	0.0436
k_s (symmetric growth rate)	hr^{-1}	0.0434	0.0434
α_1 (drug killing rate of shChk2)	hr^{-1}	0.0061	0.0133
α_2 (drug killing rate of shBim)	hr^{-1}	0.0152	0.0177
α_s (symmetric drug killing rate)	hr^{-1}	0.0111	0.0157
